# Flexible transparent electrodes based on silver nanowires synthesized via a simple method

**DOI:** 10.1098/rsos.170756

**Published:** 2017-09-20

**Authors:** De Li, Tao Han, Lei Zhang, Huai Zhang, Hui Chen

**Affiliations:** 1 State Key Laboratory of Electronic Thin Films and Integrated Devices, School of Optoelectronic Information, University of Electronic Science and Technology of China, Chengdu 610054, People's Republic of China; 2 Chongqing Engineering Research Center for Optoelectronic Materials and Devices, Research Institute for New Material Technology, Chongqing University of Arts and Sciences, Chongqing 402160, People's Republic of China

**Keywords:** silver nanowire, synthesis, transparent electrode, flexibility

## Abstract

Silver nanowires (Ag NWs) with the length of approximately 60 µm and the diameter of approximately 300 nm are prepared via a simple, cost-effective, high-yield and eco-friendly procedure under a high molar concentration ratio of silver nitrate (AgNO_3_) solution to poly(vinyl pyrrolidone) solution. The pre-synthesized Ag NWs were analysed by scanning electron microscopy, X-ray diffraction and UV–visible spectrophotometer. Furthermore, the as-prepared silver nanowires were roll-coated on the surfaces of the polyethylene terephthalate (PET) substrates. By optimizing the concentration of silver nanowire solution, the flexible Ag NW/PET transparent electrodes with a sheet resistance of 3.8 Ω sq^−1^ at a transmittance of 70% can be fabricated. The results reported in this paper provide a basis for optimizing the growth of silver nanowires and performances of transparent electrodes.

## 1. Introduction

One-dimensional metallic nanostructures, such as silver (Ag), gold (Au), copper (Cu) and so on, exhibit unusual optical, thermal, chemical and physical properties [1,2]. Of these, silver nanowire (Ag NW) is an especially attractive metal owing to its extremely high electrical conductivity in the bulk [3]. There are various applications of Ag NWs such as catalysis [4], surface-enhanced Raman scattering [5,6], photonic crystals [7], microelectronics [8] and biological nanosensors [9]. Thus, previous strategies to synthesize Ag NWs have attracted the attentions of researchers. Up to now, a number of synthetic methods have been successfully demonstrated, including template-directed and template-free. By using templates, nanowires are prepared with controlled dimensions by special shaped materials such as carbon nanotubes, DNA chains or rod-shaped micelles [10,11]. However, the manufactures of these templates are also complex and costly. On the contrary, template-free methods involve relatively simple process steps and equipment [12–15]. Considering the cost, yield and simplicity, the polyol process has achieved significant improvements [16]. The polyol process was first introduced by Fievet *et al.* [17], who successfully synthesized colloidal particles of metals and alloys such as Ag, Au and Cu. Since then, many research groups have explored different approaches and studied a series of parameters to improve the polyol process [14,18–20]. As for this method, ethylene glycol (EG) is doubling the parts of the solvent and the reducing agent [21]. In particular, Xia *et al.* made a great contribution to polyol process for the shape-controlled synthesis of Ag NWs [12–14,22–24]. They successfully synthesized Ag NWs with an average diameter of 100 nm, when the molar ratio of AgNO_3_ to poly(vinyl pyrrolidone) (PVP) was 0.67 in the presence of Pt seeds [14]. In 2007, they introduced copper (I) or copper (II) chloride into the reaction system to synthesize Ag NWs with an average diameter of 100 nm by a high molar ratio of AgNO_3_ to PVP [25]. However, silver nanowires with relatively larger diameter have not been synthesized to obtain a better conductivity.

In this paper, we report a simple method without externally added a soft template and mineral salts, by a high value of c (AgNO_3_): c (PVP) to synthesize Ag NWs with a large diameter. Furthermore, transparent conductive films were prepared by using as-synthesized Ag NWs.

## 2. Experimental section

### 2.1. Material

PVP (*M*_W_ ≈ 1 300 000) was purchased from Sigma-Aldrich. Anhydrous EG (99.8%), platinum chloride (PtCl_2_, 99.99%) and silver nitrate (AgNO_3_, 99%) were of analytical grade and purchased from Chengdu Kelong Chemical Corporation. All chemicals were used without further purification.

### 2.2. Synthesis of silver nanowires

All glassware was cleaned with deionized water, basic solution, acidic solution, acetone, isopropyl alcohol and finally deionized water. In a typical experiment, 2 ml of AgNO_3_ EG solution (in a range of 0.01–0.05 M) and 2 ml of 0.1 M PVP EG solution were put into a disposable glass, mixed and stirred evenly. Then, 10 µl of 0.038 M PtCl_2_ EG solution was injected using a syringe into the mixed solution. Afterwards, the mixed solutions were sealed and incubated at 160°C for 10 h. Finally, the precipitate was washed with deionized water five times to remove PVP residue. The products were dispersed into deionized water for further characterization.

### 2.3. Fabrication of flexible silver nanowire/polyethylene terephthalate electrodes

The Ag NWs were dispersed in ethanol, diluted to different concentrations (77, 52 and 38 mg ml^−1^) and then sonicated for 2 min to minimize the agglomeration of nanowires. The polyethylene terephthalate (PET) substrates were treated with oxygen plasma which could enhance the surface hydrophilicity in order to form uniform Ag NW networks. Then, these resulting solutions were dripped onto 300 mm × 500 mm PET substrates and pushed on top of the substrates with a Meyer rod 10#. After drying in an oven at 60°C for 30 s, additional Ag NW layers were coated on the initial Ag NW layer five times.

### 2.4. Characterizations

The micrograph samples were observed by scanning electron microscopy (SEM, Quanta 250, FEI, USA) with an acceleration voltage of 20 kV and field emission-SEM (FSEM, JSM-7800F, JEOL, Tokyo, Japan). Structure characterization were taken by an X-ray powder diffraction (XRD, TD-3500, Dandong, China) with monochromatized CuK radiation with an accelerating voltage of 30 kV and an applied current of 20 mA. The absorption spectra were recorded on a UV–vis–NIR spectrophotometer (UV-2010, G9825A, Agilent Technology, USA) using deionized water as the reference solution at room temperature. The film thickness was determined with a profilometer (KLA-Tencor, D-100, California, USA). The transmission spectra were acquired by the UV–vis–NIR spectrophotometer with a blank PET as the reference. The sheet resistance was obtained via a four point probe (RTS-5, uncommon, China).

## 3. Results and discussion

**Figure 1.**
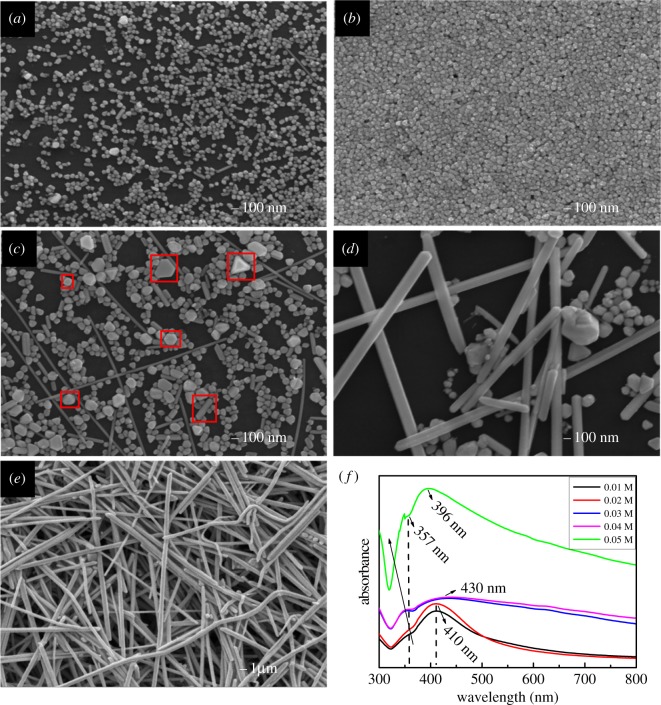
SEM images of products synthesized by adding the different c (AgNO_3_) of (*a*) 0.01, (*b*) 0.02, (*c*) 0.03, (*d*) 0.04 and (*e*) 0.05 M. (*f*) UV–visible extinction spectra of the reaction product under different molar concentration of AgNO_3_ solution.

The morphologies and sizes of products are strongly dependent on the ratio of c (AgNO_3_) : c (PVP) [14]. Figure 1*a–e* shows SEM images of products synthesized with the different molar concentration of AgNO_3_ solution. First, the Pt nanoparticles were formed by reducing PtCl_2_ with EG. With the addition of Ag^+^ ions, Ag nanoparticles are firstly formed and their average diameter increases from 50 to 65 nm via homogeneous and heterogeneous nucleation (figure 1*a*,*b*). In the process of heterogeneous nucleation, the pre-synthesized Pt nanoparticles served as nuclei for the epitaxial growth of silver due to the good match of their crystal structures and lattice parameters [14]. On the other hand, homogeneous nucleation as the other process is underway. The Ag nanoparticles are monodispersed because of the chemisorption of PVP molecules [26]. When more Ag^+^ ions were added, some Ag nanoparticles start to dissolve or grow larger via Oswald ripening with the continuation of this process [27], and Ag nanostructures with various morphologies will be produced, including spherical, cubic, triangular, polygon slab-like, rod-like and wire-like nanostructures (see red squares of figure 1*c*). The PVP molecules selectively adsorb on some facets of these particles to grow into multitwin particles. Under these conditions, rod-like Ag multitwin particles will be formed because the (100) faces of these multitwin particles are passivated by PVP and the (111) planes are active for anisotropic growth at (110) direction. However, some rod-like Ag particles could not grow longer due to the deficiency of Ag^+^ ion. As the molar concentration of AgNO_3_ solution increases to 0.04 M, an amount of Ag NWs are observed, as shown in figure 1*d*. As the molar concentration of AgNO_3_ solution increases up to 0.05 M, the single Ag NWs with the average length of approximately 60 µm and diameter of approximately 300 nm can be obtained (figure 1*e*).

The UV–vis spectroscopic method was used to characterize Ag nanostructures because of the surface plasmon resonance bands at different frequencies of different morphologies [28]. Figure 1*f* shows the UV–visible spectra obtained from the reaction product under different molar concentration of AgNO_3_ solution. Four typical absorption peaks are observed at wavelengths of approximately 357 nm, approximately 396 nm, approximately 410 nm and approximately 430 nm. With the increase of molar concentration of AgNO_3_ solution from 0.01 to 0.02 M, the absorption intensity of approximately 410 nm peak increases slightly, which indicates that the Ag nanoparticles increase in number. At the same time, the width of approximately 410 nm peak becomes larger, which could be attributed to more diverse morphologies of the Ag nanoparticles. When molar concentration of AgNO_3_ solution is further increased, there is a red shift in the maximum absorption of the spectrum, indicating that the size of Ag nanoparticle become larger. In addition, the 430 nm peak exhibits a broad full-width at half-maximum of approximately 100 nm attributed to the tailing effect, which demonstrates the existence of Ag nanoparticles with a broad distribution in size and morphology [22,29]. When the molar concentration of AgNO_3_ solution increases to 0.05 M, the absorption intensity of the maximum peak abruptly increases by more than twofold, which implies the growth rate of Ag NWs could be greatly accelerated when a critical concentration of nanorods has been reached. The maximum peak has a blue-shift from approximately 430 nm to approximately 396 nm as the average length of Ag NWs is increased [30,31]. Therefore, the peak at approximately 396 nm could be considered as the optical signature of relatively long silver nanowires [14]. Similar to the optical signatures of bulk silver, the approximately 357 nm peak exists in all five curves, which suggests that these final products should be composed of silver colloidal particles. Therefore, the morphological characteristics of reaction products which show in the UV–vis spectrum correspond well to the SEM images. As a result, the molar concentration of reactants plays an important role on the final morphologies of products.

**Figure 2.**
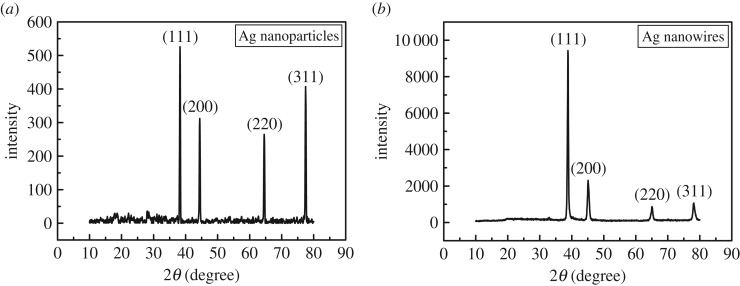
XRD patterns of as-synthesized (*a*) Ag nanoparticles and (*b*) Ag NWs.

Figure 2 depicts the XRD spectra of Ag nanoparticles and Ag NWs. The four typical peaks at 2*θ* ≈ 38.2°, 44.4°, 64.5° and 77.4° correspond to the crystalline plane of (111), (200), (220) and (311), respectively (JCPDS file No. 04-0783). In the initial stage of the process, the samples are cubic crystal structure metallic Ag nanoparticles without diffraction peaks of Ag_2_O. As the reaction process proceeds, the (111) planes of Ag particles grow actively compared with other planes. Moreover, there is a slight deviation of the four peaks at 2*θ* ≈ 38.8°, 45.1°, 65.0° and 78.0°, respectively, corresponding to the crystalline plane of (111), (200), (220) and (311) due to the growth of Ag NWs. The result is consistent with the standard diffraction spectrum of single crystal silver, indicating the face-centred cubic structure and the high crystallinity of the as-synthesized Ag NWs (figure 2*b*). The lattice constant calculated from this XRD pattern is 4.017 Å, which is very close to the theoretical data 4.0862 Å. It is worth noting that the ratio of intensity between (111) and (200) peaks exhibits a relatively high value of 4.07 (the theoretical ratio is 2.5) [14], which indicates the growth rate of (111) crystalline planes is much higher than other planes in the Ag NWs.

**Figure 3.**
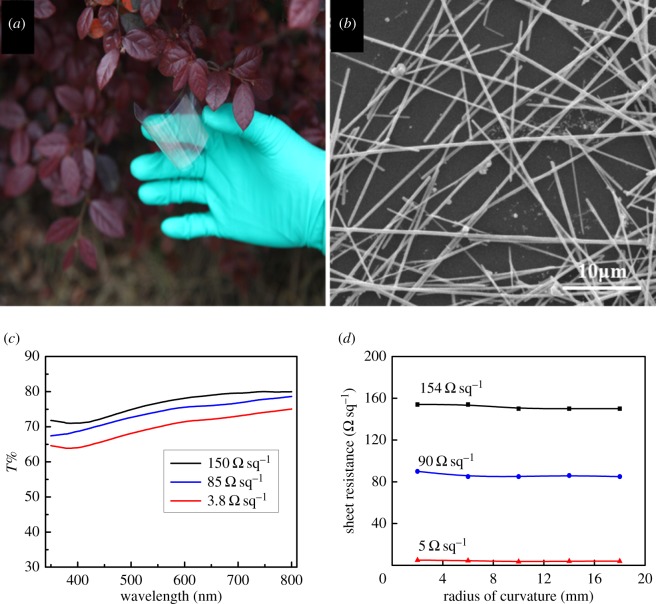
(*a*) Silver nanowire film on PET substrate with dimension of about 300 × 500 mm. (*b*) SEM image of the Ag NW/PET surface morphology. (*c*) Wavelength versus transmittance graph of Ag NW/PET transparent electrodes with different sheet resistances. (*d*) Sheet resistance versus radius of curvature for Ag NWs on PET.

Using the as-synthesized Ag NWs, flexible Ag NW/PET electrodes were fabricated. As shown in figure 3*a*, the transparent conductive film (without inert gas protection or complicated post-treatments) has an average transmittance (*T*) of 70% and a sheet resistance (*R*_sh_) of 3.8 Ω sq^−1^. The Ag NW mesh is a bit hazy because of substantial light scattering from nanowires. Figure 3*b* is the SEM image of the surface morphology of the Ag NW/PET. The Ag NWs are well interconnected on the surface of PET substrate and formed smooth conductive Ag NW networks.

The wavelength versus transmittance graph is shown in figure 3*c* for Ag NW/PET thin films. Highly conductive and transparent electrodes were fabricated such as *R*_sh_ = 3.8 Ω sq^−1^ with *T* = 70% (thickness about 2400 nm), *R*_sh_ = 85 Ω sq^−1^ with *T* = 74% (thickness about 1100 nm) and *R*_sh_ = 150 Ω sq^−1^ with *T* = 76% (thickness about 720 nm). It can be seen that the transmittance increases with the increase of sheet resistance. In general, the transmittance and sheet resistance for thin metallic films may be expressed as [32]

$$T(\lambda )={\left(1+\frac{188.5}{R_{\mathrm{sh}}}\frac{\sigma_{\mathrm{op}}(\lambda )}{\sigma_{\mathrm{DC}}}\right)}^{-2},$$

where *σ*_op_(*λ*) is the optical conductivity (here at *λ *= 550 nm) and σ_DC_ is the DC conductivity of the film. The value of *σ*_DC_/*σ*_op_(*λ*) has been used as a figure of merit for thin conducting films. In this paper, we produce thin films of Ag NWs with *σ*_DC_/*σ*_op _= 233, 13 and 8 for the data previously mentioned, respectively. The value of *σ*_DC_/*σ*_op_ at 70% transmittance (*σ*_DC_/*σ*_op _= 233) is much higher than the minimum requirement value for industry [33]. Owing to the large diameters of Ag NWs (approx. 300 nm), the light reflection and diffusion of Ag NW films increase but light transmission decreases. However, the Ag NW films as electrodes in solar cells with large reflection and scattering of light show larger photocurrent than the one using indium titanium oxide (ITO) films [34,35]. In addition, in order to demonstrate mechanical strain of the Ag NW films, the composite electrodes substrates were bent while the sheet resistance was measured (figure 3*d*). The sheet resistances of three as-roll-coated films have no significant change, even at a bending radius of 2 mm with only a slight increase. Moreover, the slight change can be attributed to scratches in the film and measurement error.

## 4. Conclusion

The process parameter c (AgNO_3_) : c (PVP) ratio for the synthesis of Ag NWs by the polyol process was studied in detail. Beyond the critical value (c (AgNO_3_): c (PVP) = 2 : 5), Ag NWs will always be synthesized and the aspect ratios of Ag NWs are constantly changing. Otherwise, the undesired Ag nanostructures with different morphologies and sizes will be synthesized. In particular, the Ag NWs with the average length of approximately 60 µm and the large average diameter approximately 300 nm were synthesized at a large ratio of c (AgNO_3_): c (PVP). Finally, the uniform and transparent thin film electrodes were fabricated via scalable, low-cost roll-coating by using as-synthesized Ag NWs. In addition, the photoelectric properties (*R*_sh_ = 3.8 Ω sq^−1^ with *T* = 70%, *R*_sh_ = 85 Ω sq^−1^ with *T* = 74% and *R*_sh_ = 150 Ω sq^−1^ with *T* = 76%) of the Ag NW electrodes were achieved. Moreover, the longer and thicker nanowires should be synthesized to increase optical transmittance with retaining the sheet resistance according to the value of σ_DC_/σ_op_. Meanwhile, the composite electrodes exhibit excellent stability of electrical property due to the slight change in bend test. Therefore, it is believed that this method will be an easy way to synthesize Ag NWs which can be applied in the field of optoelectronics, especially in organic electronic devices and solar cells.

## Data accessibility

The datasets supporting this article have been uploaded as part of the electronic supplementary material. Our data are deposited at Dryad: (http://dx.doi.org/10.5061/dryad.75r10) [36].
